# Improving the Scope of Child Mental Health Interventions in Our Modern World

**DOI:** 10.3390/ijerph20126149

**Published:** 2023-06-16

**Authors:** Barry Wright

**Affiliations:** Hull York Medical School and Department of Health Sciences, University of York, York YO10 5DD, UK; sharonbird5@nhs.net

Twenty years ago, an important systematic review showed that the empirical research evidence for interventions available for children and young people with mental health problems were rarely developed with their specific developmental needs in mind [1,2]. They were usually adaptations from treatments for adults or younger child. These researchers showed significant gaps particularly in relation to ‘attention to the biological, psychological and social dimensions of adolescent development’. This confirmed the belief of some observers that we rely too heavily on ‘adult models to guide clinical work’ for children and adolescents [3]. Years later a large comprehensive review of high-quality evidence about child mental health [4] showed that the main evidence-based interventions for depression in adolescents were cognitive behaviour therapy (CBT) (taken mainly from adult therapies) and medication (again often taken initially from adult studies). A more recent systematic review in 2020 confirmed that disappointingly little has change from the work of Weisz and Hawley all those years ago [5]. Few interventions are designed for the child and adolescent population they serve. 

This focus on adult interventions in research and clinical practice for children and young people is made more puzzling by the consistent finding that adult services and needs are very different from those of child and adolescent mental health, borne out by huge challenges in the UK around transitioning [6,7] with areas such as neurodevelopmental problems seen as of central relevance in children’s mental health services but often seen outside the remit of adult mental health services. This shows the stark difference in the perspectives towards the importance of child and adolescent development in intervention and treatment design. This is not only a UK problem; similar issues have been reported in Australia [8], the Netherlands [9], Northern Ireland [10] and beyond [11]. This gap in designing assessments and treatments specifically for the client group of children and adolescents has therefore been an international problem. This is compounded by further suggestions that clinicians and researchers often do not take into account large differences in cultural factors. While there is some high-quality child mental health treatment in some countries, a scoping review has shown that it is not readily available in low- and middle-income countries [12]. This is related to limited financial resources and policies for the provision of services, a shortage of trained interventionists and limited culturally appropriate assessment tools and treatment packages that are suitable [12,13]. Western countries also have significant challenges in availability of culturally appropriate services across a diverse society (for example in the UK [14]).

Therefore, to improve the options available therapeutically for children and young people, what therapies should we be researching? The first thing to note is that there is no reason why sitting in a room talking with an adult has to be the only way of delivering child mental health therapy. Many traumatized children and young people feel quite unsafe in this context [15]. Children who have accessed services would like more child-centred content [16] and more involvement in the choice of treatment [17,18]. Indeed, when asked, children and adolescents are clear that they would like to be more involved in the design of treatments, which, currently, rarely happens [18]. Children may respond to more child-friendly, engaging and/or appealing approaches to therapy discussed further below, and this can be the case even when the topic is serious such as in preparation for invasive examinations [19] or when the child has cancer [20,21]. 

Whilst most therapists use accessible language and terminology, this can also be a problem in some contexts [22]. Stigma is a universal challenge across the world for the provision of child mental health services [23]. The design of more child-friendly interventions integrated into mainstream environments has been identified as one of the ways of addressing this, including whole school approaches to mental health [24,25] with varied and flexible offerings [26] that provide the opportunity to normalise mental health illness prevention and make treatments more readily available. Prevention [27] and the important challenge of addressing inequalities in society [28,29] are key areas for improving the mental health of children and young people, but the focus in this editorial is on the availability of developmentally appropriate and constructive interventions that will engage children and young people. There is still relatively little evidence mainly because so little research has been funded in this area. 

There has been some encouraging research recently and the challenge does not come from the lack of ideas or early work on innovative interventions, but more in the limited number of clinical trials that would enable a treatment to enter widespread clinical practice. The challenge here, then, is for funders and commissioners. Areas of promise include interventions involving nature, play, social interactions, technology, activities, animal interactions and the arts (e.g., music, art, drama). Systematic reviews show that nature-based play in green and blue (water) spaces shows promise for child development [30] and in child mental health interventions more specifically [31] but more robust research is needed. The same is true of the use of play in therapies [32,33], play therapy more specifically [34], and creativity [35] in interventions for children, where there is much promise but little in the way of randomised controlled trials. Specific therapies have also been trialled in some groups in a limited number of contexts and many of these show positive outcomes, for example Play Brick Therapy for autistic children and young people [36]. Social prescribing has much to offer in that it appears to be helpful in conditions such as anxiety and depression [37,38] and aftercare flows more naturally after the intervention, but again there are no large randomized controlled trials to support this. In the context of pandemics, rural communities or low-income environments, consideration of the mode of delivery and settings is also important. School- and community-based interventions and digital or technological approaches may be helpful to consider and research more [39]. For example, since young people increasingly use technology, there is room to consider innovative approaches to interventions that may include computer-assisted therapy, use of websites and apps [40] and virtual reality [41] with multiple examples in this field [42,43,44,45,46] as well as video feedback interventions [47,48]. Such high tech is not, however, readily available the world over. A systematic review of child mental health interventions in low- and middle-income countries showed uses of inexpensive interventions which should be commended [49]. This includes important preventive work such as good nutrition, but also play- and classroom-based interventions. In low- and middle-income country research, authors are also suggesting more interventions that build on community and family support, and on the important work in making interventions better conceptualized for children’s needs and targeted for them specifically [50]. Interactions with animals are also an area where more research may be productive, keeping in mind the importance of the animal welfare alongside that of the child or young person [51]. Arts therapies show considerable promise. There is some limited evidence from systematic reviews that art therapy may be helpful in child mental health, for example in trauma, but there is not enough funding and studies for us to sufficiently determine its efficacy [52]. There are similar findings from systematic reviews suggesting the need for more research around music therapy [53,54,55] and drama therapy [56]. Overall, there needs to be more research funded and carried out in the field of arts psychotherapies [57]. Related to this field, storytelling and the writing of educative stories (e.g., Social Stories TM) [58] are interesting and innovative approaches.

This Special Issue sought to shine a light on some of these ideas and innovations. It contains a wide breadth of articles from new ways of conceptualizing assessment and understanding child mental health problems [59,60,61], through to descriptions of child mental health in different cultures [26,62,63] and novel ideas about treatment and care [37,64] and its mode of delivery [65]. 

The authors of the systematic reviews in the areas discussed above all advocate increased research including more research into nature-based or nature-related interventions [66], social networking interventions [67], arts therapies [57], play-based therapies [32,34], animal-assisted therapies [51] and use of technology [40]. Furthermore, the meaningful involvement of children and young people directly in the design of interventions and the delivery of research to test them, will be an important step to increase the agency of young people and to lead to better, more varied and engaging treatments [68].
